# Outcomes of Anal Cancer Patients with Inflammatory Bowel Disease Treated with Curative Chemoradiotherapy: A Systematic Review of Current Evidence

**DOI:** 10.3390/curroncol32120693

**Published:** 2025-12-09

**Authors:** Benjamin Royal-Preyra, Melanie Boucher

**Affiliations:** 1 Master of Cancer Sciences Program, University of Melbourne, Melbourne, VIC 3010, Australia; 2 Department of Radiation Oncology, Centre Hospitalier Affilié Universitaire Régional, Trois-Rivieres, QC G8Z 3R9, Canada; melanie.boucher1@ssss.gouv.qc.ca; 3 Specialized Nurse Practitioner Program-Adult Health (IPSSA), Faculty of Nursing, Université Laval, Quebec City, QC G1V 0A6, Canada

**Keywords:** anal cancer, inflammatory bowel disease, radiotherapy

## Abstract

Anal canal squamous cell cancer (ASCC) is commonly treated with chemoradiotherapy (CRT). Inflammatory bowel disease (IBD) is a relative contraindication to pelvic radiotherapy due to toxicity concerns. Advances in treatment planning since the 2000s have resulted in lower radiation doses delivered to nearby at-risk organs. We systematically reviewed the literature to answer the question “Do IBD patients with ASCC treated with CRT have different outcomes than non-IBD patients in the present era?”. We searched the Medline, Web of Science, Cochrane, ClinicalTrials.gov, and CINAHL databases for English-language articles published between 1 January 2001 and 1 January 2025. We included patients with ASCC treated curatively with CRT. This systematic review is registered in the PROSPERO international systematic review registry (CRD420251062114). A total of 220 unique articles were identified. Two reviewers independently screened titles and abstracts, which was followed by full-text screening. Eleven publications, comprising 24 patients, were included in the systematic review. The local recurrence rate following CRT was 25%, and 30% of patients had documented late toxicity. The included literature consists of case reports and small case series. Despite the limitations, our review shows toxicity, local control, and survival outcomes for IBD patients with ASCC treated with CRT that are comparable to those of non-IBD patients.

## Simple Summary

Non-metastatic anal canal cancer is usually treated with curative chemoradiotherapy. Inflammatory bowel disease (IBD) increases the risk of several gastrointestinal cancers, including anal cancer, and is a relative contraindication to pelvic radiotherapy due to potential adverse effects. Despite recent evidence showing that patients with anal cancer and IBD can be treated with pelvic radiotherapy, many patients are still treated with surgery, leading to a permanent stoma and a loss of anal continence. Technological advances since the 2000s have led to more conformal radiation plans, resulting in lower doses being delivered to organs at risk. We systematically reviewed the literature on IBD patients with anal cancer treated with curative chemoradiotherapy over the last 25 years. We found tumor control and toxicity rates that are comparable to those reported for all anal cancer patients, suggesting that non-operative treatment is safe and effective for patients with IBD.

## 1. Introduction

Anal canal cancer (AC) is an uncommon malignancy with about 50,000 new cases diagnosed and 19,000 deaths globally each year [1]. Most cases of AC are squamous cell carcinoma (ASCC), but other less common histologic types, including adenocarcinoma (5–10% of cases), lymphoma, gastrointestinal stromal tumor, melanoma, and neuroendocrine tumor, also exist [2,3]. Human papillomavirus (HPV) infection is associated with up to 85% of ASCC, with HPV subtype 16 most implicated [2,3,4]. Risk factors for ASCC include a history of anal intercourse, a high number of sexual partners, men who have sex with men, human immunodeficiency virus infection, vulva or cervix cancer or precancerous lesions, autoimmune disease, organ transplantation, inflammatory bowel disease, older age, female sex, and smoking [2,3,4].

Historically, ASCC was treated with surgery consisting of abdominoperineal resection leaving patients with a permanent colostomy [5]. Interest in the non-surgical management of ASCC with combined chemoradiotherapy (CRT) rose in the 1970s following the work by Dr. Norman Nigro [5,6]. In Dr. Nigro’s protocol, treatment consisted of 30 Gy of external beam radiotherapy (EBRT) delivered in 15 once-daily fractions of 2 Gy, 5 days per week, combined with chemotherapy consisting of 5-fluorouracil (5-FU) and mitomycin C (MMC) [5,6,7,8]. In the original protocol, CRT was planned as a preoperative treatment, and all patients were scheduled for surgery [8]. After five of the first six patients had no pathological evidence of residual disease at surgery, the protocol was changed so that surgery was only mandated in patients with residual disease four to six weeks after CRT [6,7,8]. This seminal research revealed that 84% of patients had a complete disease response (CR) to CRT and established a lack of CR after CRT as an adverse prognostic factor predictive of recurrence and death [8]. Subsequent studies evaluated the impact of changing the treatment parameters by omitting chemotherapy, changing the sequencing of chemotherapy and radiotherapy, changing the dose or the chemotherapy agents used, and altering the radiation dose and fractionation [2,9,10,11,12,13,14]. These studies confirmed the safety and efficacy of CRT for treating ASCC. Surprisingly, EBRT with concurrent 5-FU and MMC was the most effective combination, and the current treatment of ASCC patients is similar to the original Nigro protocol [2,15]. The modern treatment outcomes for ASCC vary by stage but are generally good, with approximately 70–90% 5-year overall survival and 70–86% 5-year colostomy-free survival [5]

Inflammatory bowel disease (IBD), comprising ulcerative colitis (UC) and Crohn’s disease (CD), is a chronic condition characterized by relapsing and remitting inflammation of the gastrointestinal tract [16]. Common symptoms of IBD include abdominal pain, diarrhea, rectal bleeding, weight loss, and fatigue [17]. Fistulas, fissures, and abscesses are also frequent in CD [17]. IBD is a systemic disease, and extraintestinal manifestations are common [17]. The exact cause of IBD is unknown, but it seems to involve an abnormal immune-mediated response that occurs in genetically susceptible people who are exposed to undefined environmental factors in the context of an abnormal gastrointestinal microbiome [18]. Countries in North America, Western Europe, and Northern Europe have higher rates of IBD than other regions, and these increased rates are also apparent in first-generation immigrants, suggesting that environmental factors play a crucial role in the development of IBD [19,20]. IBD is incurable, and treatment consists of medications to reduce inflammation and attenuate the immune response, with surgery used for severe refractory cases to medical management [21]. IBD increases the risk of developing gastrointestinal cancers, including colon, rectal, and ASCC [22]. A 2023 meta-analysis found ASCC rates of 10.2 and 7.7/100,000 person years for UC and CD, respectively, compared to the general population of 1.8/100,000, and this increased to 19.6 per 100,000 person years for the subset of patients with perianal CD [23].

IBD is considered a relative contraindication to abdominopelvic radiotherapy due to the fear of causing severe toxicity and is usually avoided by clinicians when alternative options exist [24,25]. Unfortunately, IBD patients are at high risk of developing malignancies for which radiotherapy is commonly used and where alternative treatments lead to significant morbidity, for example, ASCC [26]. Advances in imaging and treatment planning since the 2000s, including intensity-modulated radiation therapy (IMRT) and volumetric-modulated arc therapy (VMAT), have led to significantly more conformal radiation treatment plans with lower doses to organs at risk [27,28]. More recently published papers do not consistently report increased toxicity from radiotherapy in IBD patients and call for a more nuanced, individualized evaluation that considers IBD activity, the location of IBD relative to planned radiotherapy, and the dose to organs at risk [24,25].

The main aim of this work is to systematically review the literature and ascertain whether IBD patients treated with curative CRT for ASCC have different outcomes than non-IBD patients in the modern era.

## 2. Materials and Methods

We conducted a systematic review following the Preferred Reporting Items for Systematic Reviews and Meta-Analyses (PRISMA) 2020 framework [29] (Table S1 in the Supplementary Materials shows the PRISMA 2020 checklist).

In May 2025, we systematically searched the Medline, Web of Science, Cochrane, Scopus, ClinicalTrials.gov, and CINAHL databases for English-language literature published between 1 January 2001 and 1 January 2025. We used the keywords and Medical Subject Headings (inflammatory bowel disease or IBD or Crohn* or ulcerative colitis) AND ((anal or anus) adj3 (carcinoma* or cancer or neoplasm* or tumor* or tumor or malignan*)) AND (radiotherapy or radiation or chemo* or nonoperative or non-operative or nonsurgical or non-surgical or organ preser* or IMRT or Volumetric Arc Therapy or VMAT). Text S1 in the Supplementary Materials contains the complete search strategy used for each database. We included all study types with primary patient data. The search results were imported into Covidence systematic review software (Veritas Health Innovation, Melbourne, Australia; available at www.covidence.org).

We used the Population, Intervention, Comparison, and Outcomes framework to define study inclusion criteria (See Table 1) [30].

Two reviewers (B.R.P. and M.B.) independently screened the titles and abstracts of all articles identified by our search to identify studies that included patients with a prior diagnosis of IBD presenting with localized (stages I–III) ASCC and treated with curative-intent EBRT or CRT. Patients treated with primary surgery or where EBRT/CRT was given neoadjuvantly, adjuvantly, or with palliative intent were excluded. Title and abstract screening was performed using Covidence systematic review software (Veritas Health Innovation, Melbourne, Australia; available at www.covidence.org). A third reviewer, A.N., was available to resolve potential discrepancies between B.R.P. and M.B. Both authors reviewed the full articles that met the eligibility criteria. When eligibility was unclear based on the title and abstract, each author reviewed the full article. Both authors searched the reference lists of included articles to identify other relevant papers. Review articles without primary patient data were excluded from the systematic review; however, their reference lists were searched for other potentially eligible papers.

Reviewers assessed each study’s quality and risk of bias using the appropriate Joanna Briggs Institute (JBI) checklist [31]. Each reviewer independently assigned each study a percentage score based on the number of JBI checklist items rated “Yes”, with scores >70% considered good, 50–70% medium, and <50% low quality. All studies that met the eligibility criteria were ultimately included in the review, regardless of the quality evaluation.

The outcome domains selected to extract, and their definitions, were consistent with the International consensus to define outcomes for trials of chemoradiotherapy for anal cancer (CORMAC) core outcome set [32]. These included local/regional control, overall survival, salvage surgery, and toxicity. The authors collaborated to create a standardized electronic Excel form for data extraction. The authors extracted data independently from the included articles using the standardized electronic Excel form. The extracted data from each study included the publication year, the first author, the study type, and the sample size. Not every patient in each study met the inclusion criteria for our review; therefore, individual patients within each study were analyzed separately. Individual patient data were extracted for each patient in each study that met our review’s inclusion criteria and included the type of IBD, prior IBD treatments, length of time from IBD diagnosis to ASCC diagnosis, CRT details, acute and late toxicity following CRT, toxicity treatment, presence of recurrence of ASCC, salvage treatments for ASCC, length of follow-up, and disease and patient status at follow-up. Missing data were assumed to be missing at random (MAR) [33]. Studies were grouped and synthesized according to the type of inflammatory bowel disease (ulcerative colitis versus Crohn’s disease).

We registered this systematic review in the National Institute for Health and Care Research PROSPERO international systematic review registry (CRD420251062114, https://www.crd.york.ac.uk/PROSPERO/view/CRD420251062114 (accessed on 28 May 2025)). A review protocol is available from the authors upon request. Section 3 shows the template data collection forms and extracted patient data.

## 3. Results

**Figure 1:**
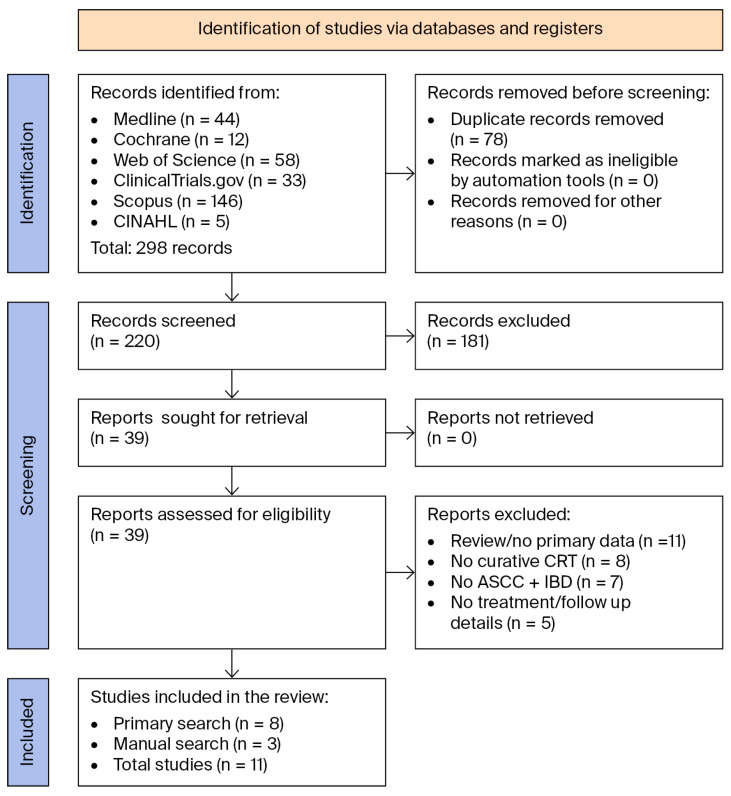
PRISMA 2020 flow diagram showing the search and screening process [29].

Figure 1 below shows the PRISMA flow diagram of our screening process [29]. Table 2 and Table 3 show the study and patient characteristics of the articles included in this review.

Our search identified 298 articles, of which 220 were screened, leaving 39 articles for full review. After a thorough review, 31 articles were excluded, namely, 11 review articles that lacked primary patient data, 8 articles that did not treat patients with curative CRT, 7 articles that did not include patients with co-occurring ASCC and IBD, and 5 articles that lacked treatment or follow-up details. Eight publications met the eligibility criteria. A manual review of the complete reference lists from the 8 eligible articles and 11 review articles identified 3 additional publications that met the eligibility criteria and 1 review article without primary patient data. The complete reference lists of these articles were also reviewed, identifying no new publications of interest. Table 1 shows the eleven publications, comprising 24 patients from seven case reports and four retrospective chart reviews that were included in the final systematic review [34,35,36,37,38,39,40,41,42,43,44].

**Table 2:** Characteristics of the studies included in our review.

Author	Year	Country	Study Type	Level of Evidence [45]	# Pts Eligible for Inclusion/Total Patients in Article	Pt # in Table 3	IBD Type
Schaffzin, D et al. [34]	2005	USA	Case report	4d	1/1	1	UC
Devon, K et al. [35]	2009	CAN	Chart review	4d	1/14	2	CD
Macdonald, E et al. [36]	2010	GBR	Case report	4d	1/1	3	UC
Shwaartz, C et al. [37]	2016	USA	Chart review	4c	6/19	4–9	CD
Lightner, A et al. [38]	2017	USA	Chart review	4c	5/7	10–14	CD
Pellino, G et al. [39]	2017	GBR	Case report	4d	1/1	15	UC
Rohrbach, S et al. [40]	2018	USA	Case report	4d	1/1	16	UC
Makowsky, M et al. [41]	2019	CAN	Case report	4d	1/1	17	CD
Weingarden, A et al. [42]	2020	USA	Case report	4d	1/1	18	CD
Sakanaka, K et al. [43]	2021	JAP	Case report	4d	1/1	19	CD
Lightner, A et al. [44]	2021	USA	Chart review	4c	5/17	20–24	UC

Abbreviations: USA; United States of America. CAN; Canada. GBR; United Kingdom of Great Britain and Northern Ireland. JPN; Japan. IBD; inflammatory bowel disease; UC; ulcerative colitis. CD; Crohn’s disease. #; number. Pt; patient. Joanna Briggs Institute Levels of Evidence 4c; case series, 4d; case reports [45].

**Table 3:** Individual patient data from publications included in our review.

Patient Number (Ref)	Age	Sex	IBD Type	Prior Medical Tx	Prior Surgical Tx	ASCC Stage	ASCC Tx	Acute Toxicity	Late Toxicity	Toxicity Tx	Outcome	FU (Months)	Status at FU	Comments
1 [34]	47	F	UC	Yes (no details)	Prophylactic ileal pouch pull-through with mucosectomy	3 × 3 cm, N0M0 on CT (stage 2)	50.4 Gy + 5FU/MMC	20 loose BMs daily	2–4 semisolid BMs daily	Lomotil x 8 daily	CR	n/a	n/a	Increased BMs preceded CRT
2 [35]	46	F	CD	n/a	n/a	Stage 2	Primary RT (no details)	n/a	n/a	n/a	RD, salvage APR	37	AWD	
3 [36]	41	M	UC	n/a	Restorative proctocolectomy, ileal s-pouch, balloon dilatation × 6, stricturoplasty, polyp excision, pouch excision	Sacrum invaded on CT/TEP (? T4N0M0, stage 3)	Local RT (dose n/a) + 5FU/MMC	n/a	n/a	n/a	RD, salvage APR not possible	12	Deceased	
4 [37]	64	M	CD	Anti-TNF	n/a	n/a	Nigro protocol	n/a	n/a	n/a	CR	>36	NED	
5 [37]	50	M	CD	Anti-TNF	n/a	n/a	Nigro protocol	n/a	n/a	n/a	CR	>36	NED	
6 [37]	45	F	CD	n/a	n/a	n/a	Nigro protocol	n/a	n/a	n/a	RD, salvage APR	>36	NED	
7 [37]	46	M	CD	Anti-TNF	n/a	n/a	Nigro protocol	n/a	n/a	n/a	RD, salvage APR	>36	NED	
8 [37]	65	F	CD	n/a	n/a	n/a	Nigro protocol	n/a	n/a	n/a	CR	>36	NED	
9 [37]	31	F	CD	Anti-TNF	n/a	n/a	Nigro protocol	n/a	n/a	n/a	CR	>36	NED	
10 [38]	49	F	CD	n/a	No	Stage 2	55 Gy + 5FU/MMC	n/a	Anal and vaginal stenosis/fibrosis 1 year post-CRT	Dilatation	CR	30 (median)	NED	
11 [38]	29	F	CD	n/a	IPAA (presumed UC)	Stage 3	50 Gy + 5FU/MMC	Anovaginal fistula and anal stricture within 2 months CRT	n/a	n/a	CR	30 (median)	NED	
12 [38]	51	F	CD	Prednisone, certolizumab	Several small bowel resections	Stage 3	55 Gy + 5FU/MMC	n/a	Intractable pain	APR/VRAM for pain (no cancer recurrence)	CR	30 (median)	NED	
13 [38]	52	F	CD	6-MP	No	Stage 1	RT (dose n/a) + 5FU/MMC	n/a	n/a	n/a	CR	30 (median)	NED	
14 [38]	28	M	CD	MTX	Seton insertion	n/a	55 Gy + 5FU/cisplatin	n/a	n/a	n/a	RD, salvage APR 12 months post-CRT	30 (median)	NED	Cisplatin due to co-occurring Hodgkin’s lymphoma
15 [39]	84	M	UC	Steroid refractory	IPAA, loop ileostomy	T1N0M0 (stage I)	54 Gy/30 fractions + 5FU/MMC	Pain and skin ulceration	Stricture/inflammation at pouch inlet	Loperamide, endoscopic balloon dilatation	CR	24	NED	
16 [40]	53	F	UC	n/a	Total colectomy, J pouch ileoanal anastomosis, recurrent pouch inflammation	Stage 3B	Chemoradiation	n/a	n/a	n/a	CR	12	NED	
17 [41]	61	F	CD	n/a	4 prior bowel resections	n/a	CRT 30 fractions + 5FU/MMC	Hospitalized at 18th fraction for pain, metabolic abnormalities due to high output ileostomy	High ostomy output causing metabolic abnormalities, radiation enteritis, bowel tethering, abnormal bowel motility related to prior surgeries, bacterial overgrowth, peri-anal wound	Several hospitalizations. PO/IV/SC Mg, hydration, loperamide, opioids. Peri-anal wound closure.	Cancer outcome not explicitly mentioned (? NED)	31	Alive, off Mg supplements (cancer status not mentioned)	
18 [42]		M	CD	Ileal release budenoside, AZA, infliximab, MTX, adalimumab, 6-MP, mesalamine	n/a	n/a, possible T4N0	CRT (no details)	n/a	Peri-anal fistula (post-salvage APR)	Abx, fistula drainage	RD, salvage APR 20 months post-CRT. RD post APR. Treated with palliative chemotherapy	22	? AWD (unclear)	Fistulizing peri-anal CD pre-dated CRT
19 [43]		M	CD	Total parenteral nutrition, immunosuppressants, mesalamine, 5-ASA, AZA, infliximab	Temporal ileostomy	Stage 2B	15 MV photons, VMAT, 41.4 Gy/23 fractions, boost to 54 Gy, + 5FU/MMC	G3 leukopenia, G3 neutropenia, G2 anemia, G1 thrombocytopenia, G2 appetite loss, G2 diarrhea, G2 dermatitis, G2 anal pain	Ileus 14 months post-CRT	Conservative management	CR	24	NED	
20 [44]		n/a	UC	AZA	No	Stage 2	45–60 Gy RT + 5FU/MMC	n/a	n/a	n/a	CR	60	NED	
21 [44]		n/a	UC	Corticosteroids, 6-MP	Ileorectal-anastomosis	Stage 2	45–60 Gy RT + 5FU/MMC	n/a	n/a	n/a	CR	60	NED	
22 [44]		n/a	UC	Corticosteroids	IPAA, diversion 10–20 days before CRT	Stage 3	45–60 Gy RT + 5FU/MMC	n/a	n/a	n/a	CR	60	NED	Diversion pre-CRT
23 [44]		n/a	UC	n/a	IPAA, diversion 10–20 days before CRT	Stage 3	45–60 Gy RT + 5FU/MMC	n/a	n/a	n/a	CR	60	NED	Diversion pre-CRT
24 [44]		n/a	UC	n/a	IPAA	Stage 3	45–60 Gy RT + 5FU/MMC	MI during CRT, 40 BMs daily, incontinence	n/a	Diversion and pouch excision during treatment	Died during CRT from MI	0	Deceased	
Total		Female: 45.8% (*n* = 11) Male: 33.3% (*n* = 8) n/a: 20.8% (*n* = 5)	CD: 62.5% (*n* = 15) UC: 37.5% (*n* = 9)	Yes: 58.3% (*n* = 14) No: 0% (*n* = 0) n/a: 41.7% (*n* = 1)	Yes: 54.2% (*n* = 13) No: 12.5% (*n* = 3) No n/a: 33.3% (n = 8)	Stage 1: 8.3% (*n* = 2) Stage 2: 25% (*n* = 6) Stage 3: 29.2% (*n* = 7) n/a: 37.5% (*n* = 9)	CRT: 95.8% (*n* = 23) RT alone: 4.2% (*n* = 1)	24% (*n* = 6) of patients with documented acute toxicity	29.2% (*n* = 7) of patients with documented late toxicity		CR: 66.7% (*n* = 16) RD: 25% (*n* = 6) Unclear: 4.2% (*n* = 1) Died during CRT: 4.2% (*n* = 1)	Median: 30.5-month follow-up	NED: 75% (*n* = 18) AWD: 4.2% (*n* = 1) NED/AWD (unclear): 8.3% (*n* = 2) Deceased: 8.3% (*n* = 2) n/a): 4.2% (*n* = 1)	

Abbreviations: *n*; number of people. ASCC; squamous cell anal cancer. CRT; chemoradiotherapy. FU; follow-up. UC; ulcerative colitis. CD; Crohn’s disease. n/a; not reported by study authors. Gy; gray. 5FU; fluorouracil. MMC; mitomycin C. BMs; bowel movements. NED; no evidence of disease. RD; residual disease. APR; abdominoperineal resection. AWD; alive with disease. TNF; tumor necrosis factor. IPAA; ileal pouch anal anastomosis. MV; megavoltage. VMAT; volumetric modulated arc therapy. CR; complete response. MI; myocardial infarction. CT; computed tomography scan. AZA; azathioprine. MTX; methotrexate. 5-ASA; mesalamine. 6-MP; mercaptopurine. Abx; antibiotics. Tx; Treatment. CD disease patients with ASCC.

### 3.1. CD Patients with ASCC

Our search identified 15 patients with CD and ASCC from three retrospective chart reviews and three case reports [35,37,38,41,42,43]. The mean age at diagnosis of ASCC was 49 (range 28–65), and 60% (*n* = 9) were female. The most common reported presenting ASCC symptom was anal pain or irritation in 40% of patients (*n* = 6) and worsening anal drainage or seepage in 26.7% (*n* = 4). The presenting symptoms were not reported in 40% (*n* = 6) of patients. The ASCC stage was not reported in 60% (*n* = 9) of cases. In the 6 patients with stage information available, the distribution was 16.7% in stage I (*n* = 1), 50% in stage II (*n* = 3), and 33.3% in stage III (*n* = 2).

### 3.2. UC Patients with ASCC

Our search identified nine patients with UC and ASCC from one retrospective chart review and four case reports [34,36,39,40,44]. The mean age at ASCC diagnosis was 57.9 years (range 41–84 years). In studies that reported patient sex, 22.2% (*n* = 2) were male, and 22.2% (*n* = 2) were female. Sex was unknown for 55.6% (*n* = 5) of patients. The most common presenting symptoms of ASCC were rectal bleeding 66.7% (*n* = 6), pain or irritation 33.3% (*n* = 3), and anal drainage or seepage 22.2% (*n* = 2). The distribution of stage at ASCC diagnosis was as follows: 11.1% stage I (*n* = 1), 33.3% stage II (*n* = 3), and 55.6% stage III (*n* = 5).

### 3.3. Differences Between CD and UC Patients

A total of 86.7% (*n* = 13) of CD patients with ASCC had a history of perianal disease compared to 11.1% (*n* = 1) of UC patients. In contrast, 88.9% (*n* = 8) of UC patients had a history of surgical interventions for IBD compared to only 20% (*n* = 3) for CD patients. Most UC patients (66.7%) presented with rectal bleeding as a presenting symptom of ASCC, whereas this was not reported for CD patients. Worsening anal pain and drainage were common presenting symptoms in both UC and CD patients with ASCC.

### 3.4. Similarities Between CD and UC Patients

IBD preceded the diagnosis of ASCC by approximately two decades for both CD (median 22 years) and UC (median 22.6 years). In the months leading to ASCC diagnosis, 26.6% (*n* = 4) of CD and 44.4% (*n* = 4) of UC patients with ASCC experienced nondiagnostic imaging, nonconfirmatory biopsies, or management for presumed IBD-related symptoms, potentially delaying treatment.

### 3.5. ASCC Treatment

Almost all, or 93.3% (*n* = 14), of patients with CD were treated with CRT, and one patient was treated with primary radiation alone. All UC patients were treated with CRT. Concurrent chemotherapy was 5FU/MMC for 83.3% (*n* = 20) of all patients. Doses ranged between 45 and 60 Gy of conventionally fractionated external beam radiotherapy in the 79.2% of patients with a reported radiation dose.

### 3.6. Toxicity from ASCC Treatment

Overall, 25% (*n* = 6) of patients developed acute toxicity to CRT, including 20% (*n* = 3) of the 15 CD patients and 33.3% (*n* = 3) of the 9 UC patients. One patient had increased stool frequency (up to 40 bowel movements daily) that necessitated IPAA and pouch excision during CRT and died of a myocardial infarction during treatment [44]. Two other patients did not experience acute toxicity from CRT per se but had undergone fecal diversion before CRT due to concerns over potential toxicity [44]. Late toxicity was seen in 29.2% (*n* = 7) of all patients, including 33.3% (*n* = 5) of the CD patients and 22.2% (*n* = 2) of UC patients.

### 3.7. Local Control and Survival Outcomes

With a median follow-up of 31 months, local control was approximately 66.7% (*n* = 10) for CD patients. One-third (*n* = 5) of CD patients recurred and were treated with salvage APR. All CD patients were alive at the time of follow-up, 86.7% (*n* = 13) with no evidence of disease (NED) and 13.3% (*n* = 2) alive with disease (AWD).

With a median follow-up of 24 months, local failure was reported in only 11.1% (*n* = 1) of UC patients. Most or 77.8% (*n* = 7) of UC patients were alive at follow-up. In the largest series that included five patients with UC treated for ASCC, 80% (*n* = 4) of patients were alive with no evidence of disease at 5-year follow-up [44]. One UC patient died from a myocardial infarction during CRT, and one died from recurrent ASCC 12 months post-CRT [36,44].

### 3.8. Level of Evidence and Study Quality

Each study was critically appraised using the Joanna Briggs Institute (JBI) tools for case reports and case series (see Table 4 below) [31,45,46]. Good quality was defined as having >70% of checklist items completed, medium quality as 50–70%, and low quality as <50%. Over half (*n* = 6) of the studies were rated as good quality and the remainder as medium quality. Common missing items included incomplete CRT details in 45.5% (*n* = 5) of studies, inadequate follow-up information in 27.3% (*n* = 3), missing ASCC staging in 27.3% (*n* = 3), and inadequate reporting of adverse effects in 27.3% (*n* = 3). All studies were case reports or case series and had JBI levels of evidence of 4d or 4c, respectively [45].

## 4. Discussion

To our knowledge, this is the first systematic review aimed explicitly at characterizing the outcomes of IBD patients with ASCC treated nonoperatively in the modern era. The publication dates spanned 16 years, and all papers except one were from the United States, Canada, or the United Kingdom. These studies show that IBD patients with ASCC treated with CRT have toxicity, local control, and survival outcomes that are comparable to those of patients without IBD.

IBD patients with ASCC might present with more advanced stages of cancer compared to their non-IBD counterparts [24]. About half of all ASCC patients present with localized disease, a third with disease that has spread to the regional lymphatics, and approximately ten percent present with metastatic disease [47]. Only 62.5% (*n* = 15) of patients in our review had stage documented; however, 46.7% (*n* = 7) of these patients presented with stage III disease. Possible reasons that IBD patients might present with more advanced ASCC include diagnostic delays due to IBD and ASCC presenting similarly, IBD patients often being on long-term immunosuppressive medications, or possible inherent imaging and pathologic differences between IBD and non-IBD patients [35]. One-third of the patients in our review experienced diagnostic delays, including inconclusive biopsies [34,35,36], nondiagnostic imaging [35], repeated colonoscopies [42], and treatment for perianal CD symptoms [41,42,43] in the months leading up to ASCC diagnosis.

Approximately one-quarter and one-third of patients had documented acute and late toxicity, respectively, in this review. Toxicity grading was reported in only one study, making comparisons with other studies difficult. It is also probable that toxicity was underreported in these studies and that only adverse effects considered by the authors to be significant were mentioned. In contrast, the ACT II trial reported 71% grade 3 or 4 toxicity (any) for patients treated with CRT [13]. In our case series, four patients (16.7%) had increased stool frequency, and two required hospitalization during CRT, compared with only 4% in the ACT II trial. However, it is worth mentioning that all the patients in our review with severe increased stool frequency during CRT had a history of gastrointestinal surgeries (sometimes multiple) for IBD. This toxicity was not reported for patients without a prior history of IBD-related surgery. Thus, these patients might represent a subset of patients at higher risk of CRT toxicity.

The patients in this review had more treatment-related deaths than the 2% and <1% reported in the ACT I and II trials, respectively [13,48]. One patient (4.2% of all patients) in this case series died during CRT [44]. This was a 62-year-old man with a history of prior IPAA for UC who died two months into CRT from MI. The reason for his death is likely multifactorial and cannot be attributed solely to his pelvic radiotherapy. This patient also underwent fecal diversion and pouch excision either immediately before CRT or during CRT (the article does not specify), and while his treatment with CRT was likely a factor in his myocardial infarction, attributing this solely to a “radiation-induced myocardial infarction” while ignoring his recent abdominal surgery and ongoing chemotherapy does not provide a complete picture. Patients with IBD and ASCC may be at greater risk of treatment-related deaths than non-IBD patients, but the death seen in this review could also represent a spurious finding and should be interpreted with caution.

In this review, 25% of patients (*n* = 6) recurred following CRT, compared to 31.2% reported for risk of locoregional relapse and 73% reported for 3-year progression-free survival in the ACT I and II trials, respectively [13,48]. All CD patients and 77.8% (*n* = 7) of patients with UC were alive at last follow-up. In the largest UC series, which included five patients treated with CRT, 80% were alive with no evidence of disease five years after CRT, which is consistent with the 5-year survival rates of 83% and 67% for localized and regional ASCC in the Surveillance, Epidemiology, and End Results (SEER) data [49].

IBD preceded an ASCC diagnosis by over two decades in this review. Future research could analyze whether more aggressive management of IBD-related gastrointestinal inflammation could decrease cancer incidence; however, the relative harms and benefits of this strategy are not obvious given that many medications for treatment of IBD cause immunosuppression, which is itself a risk factor for developing cancers [50]. HPV vaccination programs will likely reduce ASCC in IBD patients in the coming decades, as HPV is also a significant etiologic agent in this population [51,52]. Enhanced use of HPV screening programs for IBD patients to facilitate early identification of pre-cancerous lesions or early anal cancers might improve outcomes by reducing the diagnostic delays that this population experiences [51,52]

This review has many limitations. The articles included in the review were all case reports and small case series, which are prone to selection, publication, and confirmation bias [53,54]. Furthermore, the search strategy was limited to English-language papers from six databases, potentially biasing the publications toward Western populations. It is unclear whether these findings can be generalized to all patients with IBD and ASCC. Staging, treatment, toxicity, and follow-up data were incomplete in many articles, making it challenging to compare the patients in this review to the published ASCC literature. Nevertheless, the data suggest that IBD patients with ASCC treated nonoperatively have broadly comparable outcomes to non-IBD patients. Further research is needed to determine whether some patients are better treated with surgery than with nonoperative management, and whether nonoperative management should remain the standard approach for most patients.

## 5. Conclusions

This systematic review shows that the toxicity, local control, and survival outcomes for patients with IBD and ASCC treated nonoperatively are comparable to non-IBD patients. Patients with a history of surgical treatments for IBD might be at higher risk for gastrointestinal toxicity from CRT. A higher percentage of treatment-related deaths than expected was observed in this review, but this represents only one patient and is of unclear significance. The diagnosis of IBD preceded ASCC by over 20 years in both UC and CD patients in this review. A significant number of patients faced delays in diagnosis due not only to ASCC symptoms initially being attributed to IBD but also because of nondiagnostic imaging and biopsies, potentially contributing to a more advanced ASCC stage at diagnosis. Future research characterizing how imaging and pathologic features differ between IBD patients with ASCC and non-IBD patients could be conducted to help identify patients with ASCC at earlier stages. This review consists of case reports and small case series, making generalization difficult. This review could influence multidisciplinary decision-making and patient counseling for IBD patients with anal cancer. Judicious patient evaluation that considers individual patients’ IBD characteristics, and high-quality radiotherapy treatment planning with particular attention to organ-at-risk dose constraints, could help clinicians select patients most likely to have a good outcome with non-operative treatment.

## Figures and Tables

**Table 1:** Study eligibility criteria.

Criteria	Inclusion	Exclusion
Population	Patients with IBD and ASCC *	Patients with metastatic disease
Intervention	Treatment with curative EBRT or CRT	Neoadjuvant EBRT/CRT Adjuvant EBRT/CRT Palliative intent treatment Salvage CRT Brachytherapy
Comparison	ASCC patients without IBD treated with curative EBRT or CRT	
Outcomes	Acute and late toxicity, local control, and survival following CRT	
Study type	Case reports, case series, randomized controlled trials, abstracts, reviews, and meta-analyses **	Studies without primary patient data. Studies without outcomes reported.
Inclusion dates	Patients treated between 1 January 2001 and 1 January 2025	

* Distal rectum/transition zone squamous cell carcinomas were included; ** Review articles were used to identify publications with primary patient data.

**Table 4:** Critical appraisal using Joanna Briggs Institute Checklists.

Study	% Yes	No/Unclear	Quality	Level of Evidence	Comments
Schaffzin, D et al. [34]	62.5%	37.5%	Medium	4d	No follow-up reported
Devon, K et al. [35]	50%	50%	Medium	4d (case series, but only 1 patient eligible for this review)	Missing RT and toxicity details, prior IBD treatments
MacDonald, E et al. [36]	50%	50%	Medium	4d	Missing RT and toxicity details
Shwaartz, C [37]	80%	20%	Good	4c	No stage reported. Lack of CRT details
Lightner, A [38]	100%	0%	Good	4c	
Pellino, G et al. [39]	100%	0%	Good	4d	
Rohrbach, S et al. [40]	75%	25%	Good	4d	No CRT or toxicity details
Makowsky, M et al. [41]	62.5%	37.5%	Medium	4d	CRT details incomplete. ASCC status unclear
Weingarden, A et al. [42]	62.5%	37.5%	Medium	4d	Unclear ASCC stage pre-CRT. No CRT details. Unclear ASCC outcome
Sakanaka, K et al. [43]	100%	0	Good	4d	
Lightner, A et al. [44]	80%	20%	Good	4c	Sex of cases not reported. Pre-CRT surgery and MI counted as acute radiotoxicity

Abbreviations: RT; radiotherapy. IBD; inflammatory bowel disease. CRT; chemoradiotherapy. ASCC; anal squamous cell carcinoma. MI; myocardial infarction. Joanna Briggs Institute Levels of Evidence 4c; case series, 4d; case reports [45].

## Data Availability

All data are found in the article and Supplementary Materials.

## Supplementary Materials

The following supporting information can be downloaded at https://www.mdpi.com/article/10.3390/curroncol32120693/s1, Table S1: PRISMA 2020 Checklist; Text S1: Database search strategy.
