# Supplementary material for: Outcomes of Anal Cancer Patients with Inflammatory Bowel Disease Treated with Curative Chemoradiotherapy: A Systematic Review of Current Evidence
Source: Curr Oncol. 2025 Dec 9;32(12):693. doi: 10.3390/curroncol32120693 (PMC12731345; doi:10.3390/curroncol32120693)
Supplement: Supplementary file 1 [file curroncol-32-00693-s001.zip › curroncol-3979228-supplementary.pdf]

Table S1. PRISMA 2020 Checklist.

Section and Topic	Item #	Checklist item	Location where item is reported
TITLE			
Title	1	Identify the report as a systematic review.	Line 1
ABSTRACT			
Abstract	2	See the PRISMA 2020 for Abstracts checklist.	Line 26-44
INTRODUCTION			
Rationale	3	Describe the rationale for the review in the context of existing knowledge.	Line 96-104
Objectives	4	Provide an explicit statement of the objective(s) or question(s) the review addresses.	Line 106-107
METHODS			
Eligibility criteria	5	Specify the inclusion and exclusion criteria for the review and how studies were grouped for the syntheses.	Table 1, Line 130-133
Information sources	6	Specify all databases, registers, websites, organisations, reference lists and other sources searched or consulted to identify studies. Specify the date when each source was last searched or consulted.	Line 114-116
Search strategy	7	Present the full search strategies for all databases, registers and websites, including any filters and limits used.	S2 supplementary materials
Selection process	8	Specify the methods used to decide whether a study met the inclusion criteria of the review, including how many reviewers screened each record and each report retrieved, whether they worked independently, and if applicable, details of automation tools used in the process.	Line 130-140
Data collection process	9	Specify the methods used to collect data from reports, including how many reviewers collected data from each report, whether they worked independently, any processes for obtaining or confirming data from study investigators, and if applicable, details of automation tools used in the process.	Line 141-158
Data items	10a	List and define all outcomes for which data were sought. Specify whether all results that were compatible with each outcome domain in each study were sought (e.g. for all measures, time points, analyses), and if not, the methods used to decide which results to collect.	Line 141-158
	10b	List and define all other variables for which data were sought (e.g. participant and intervention characteristics, funding sources). Describe any assumptions made about any missing or unclear information.	Line 146-158
Study risk of bias assessment	11	Specify the methods used to assess risk of bias in the included studies, including details of the tool(s) used, how many reviewers assessed each study and whether they worked independently, and if applicable, details of automation tools used in the process.	Line 141-145
Effect measures	12	Specify for each outcome the effect measure(s) (e.g. risk ratio, mean difference) used in the synthesis or presentation of results.	Not applicable

Section and Topic	Item #	Checklist item	Location where item is reported
Synthesis methods	13a	Describe the processes used to decide which studies were eligible for each synthesis (e.g. tabulating the study intervention characteristics and comparing against the planned groups for each synthesis (item #5)).	Not applicable
	13b	Describe any methods required to prepare the data for presentation or synthesis, such as handling of missing summary statistics, or data conversions.	Not applicable
	13c	Describe any methods used to tabulate or visually display results of individual studies and syntheses.	Not applicable
	13d	Describe any methods used to synthesize results and provide a rationale for the choice(s). If meta-analysis was performed, describe the model(s), method(s) to identify the presence and extent of statistical heterogeneity, and software package(s) used.	Not applicable
	13e	Describe any methods used to explore possible causes of heterogeneity among study results (e.g. subgroup analysis, meta-regression).	Not applicable
	13f	Describe any sensitivity analyses conducted to assess robustness of the synthesized results.	Not applicable
Reporting bias assessment	14	Describe any methods used to assess risk of bias due to missing results in a synthesis (arising from reporting biases).	Line 157
Certainty assessment	15	Describe any methods used to assess certainty (or confidence) in the body of evidence for an outcome.	Not applicable
RESULTS			
Study selection	16a	Describe the results of the search and selection process, from the number of records identified in the search to the number of studies included in the review, ideally using a flow diagram.	Figure 1, Line 167-177
	16b	Cite studies that might appear to meet the inclusion criteria, but which were excluded, and explain why they were excluded.	Figure 1, Line 169-174
Study characteristics	17	Cite each included study and present its characteristics.	Tables 2 and 3
Risk of bias in studies	18	Present assessments of risk of bias for each included study.	Table 4, Line 252-257
Results of individual studies	19	For all outcomes, present, for each study: (a) summary statistics for each group (where appropriate) and (b) an effect estimate and its precision (e.g. confidence/credible interval), ideally using structured tables or plots.	Table 3
Results of syntheses	20a	For each synthesis, briefly summarise the characteristics and risk of bias among contributing studies.	Not applicable
	20b	Present results of all statistical syntheses conducted. If meta-analysis was done, present for each the summary estimate and its precision (e.g. confidence/credible interval) and measures of statistical heterogeneity. If comparing groups, describe the direction of the effect.	Not applicable
	20c	Present results of all investigations of possible causes of heterogeneity among study results.	Not applicable

Section and Topic	Item #	Checklist item	Location where item is reported
	20d	Present results of all sensitivity analyses conducted to assess the robustness of the synthesized results.	Not applicable
Reporting biases	21	Present assessments of risk of bias due to missing results (arising from reporting biases) for each synthesis assessed.	Not applicable
Certainty of evidence	22	Present assessments of certainty (or confidence) in the body of evidence for each outcome assessed.	Not applicable
DISCUSSION			
Discussion	23a	Provide a general interpretation of the results in the context of other evidence.	Line 265-266
	23b	Discuss any limitations of the evidence included in the review.	Line 302-306
	23c	Discuss any limitations of the review processes used.	Line 303-304
	23d	Discuss implications of the results for practice, policy, and future research.	Line 306-307
OTHER INFORMATION			
Registration and protocol	24a	Provide registration information for the review, including register name and registration number, or state that the review was not registered.	Line 159-161
	24b	Indicate where the review protocol can be accessed, or state that a protocol was not prepared.	Line 161
	24c	Describe and explain any amendments to information provided at registration or in the protocol.	Not applicable
Support	25	Describe sources of financial or non-financial support for the review, and the role of the funders or sponsors in the review.	Line 324
Competing interests	26	Declare any competing interests of review authors.	Line 331
Availability of data, code and other materials	27	Report which of the following are publicly available and where they can be found: template data collection forms; data extracted from included studies; data used for all analyses; analytic code; any other materials used in the review.	Table 2 and 3, Line 161

Text S1: Database search strategy

Medline

Ovid MEDLINE(R) ALL <1946 to February 24, 2025>

1 exp Inflammatory Bowel Diseases/ 104772

2 (Inflammatory and Bowel and Disease*).mp. 83287

3 (IBD or Crohn* or ulcerative colitis).mp. 123597

4 1 or 2 or 3 157220

5 ((anal or anus) adj3 (carcinoma* or Adenocarcinoma or cancer* or neoplasm* or tumour* or tumor* or malignan* or squamous or melanoma or small cell)).mp. 10659

6 (Non-operative or nonoperative or Radiotherapy or Radiation or chemotherapy or Chemo* or Nonsurgical or Non-surgical or organ perserv* or IMRT or Volumetric Modulated Arc Therapy or VMAT).mp. 1838001

Web of Science

1: ALL=((Inflammatory Bowel Disease* OR IBD OR Crohn* OR ulcerative colitis)) Results: 232969

2: TS=((anal OR anus) NEAR/3 (carcinoma* OR adenocarcinoma OR cancer* OR neoplasm* OR tumour* OR tumor* OR malignan* OR squamous OR melanoma OR "small cell")) Results: 9877

3: ALL=(Non-operative OR nonoperative OR Radiotherapy OR Radiation OR chemotherapy OR Chemo* OR Nonsurgical OR Non-surgical OR organ perserv* OR IMRT OR Volumetric Modulated Arc Therapy OR VMAT) Results: 3017254

4: #3 AND #2 AND #1 Results: 58

Cochrane:

EBM Reviews - Cochrane Database of Systematic Reviews <2005 to February 19, 2025>
EBM Reviews - ACP Journal Club <1991 to January 2025>
EBM Reviews - Database of Abstracts of Reviews of Effects <1st Quarter 2016>
EBM Reviews - Cochrane Clinical Answers <February 2025>
EBM Reviews - Cochrane Central Register of Controlled Trials <December 2024>
EBM Reviews - Cochrane Methodology Register <3rd Quarter 2012>
EBM Reviews - Health Technology Assessment <4th Quarter 2016>
EBM Reviews - NHS Economic Evaluation Database <1st Quarter 2016>

1 exp Inflammatory Bowel Diseases/ 5159
2 (Inflammatory and Bowel and Disease*).mp. 6053
3 (IBD or Crohn* or ulcerative colitis).mp. 13365
4 1 or 2 or 3 15589
5 ((anal or anus) adj3 (carcinoma* or Adenocarcinoma or cancer* or neoplasm* or tumour* or tumor* or malignan* or squamous or melanoma or small cell)).mp. 834
6 (Non-operative or nonoperative or Radiotherapy or Radiation or chemotherapy or Chemo* or Nonsurgical or Non-surgical or organ perserv* or IMRT or Volumetric Modulated Arc Therapy or VMAT).mp. 169039
7 4 and 5 and 6 12

SCOPUS

(TITLE-ABS-KEY ((non-operative OR nonoperative OR radiotherapy OR radiation OR chemotherapy OR chemo* OR nonsurgical OR non-surgical OR "organ preserv*" OR imrt OR "volumetric modulated arc therapy" OR vmat))) AND (TITLE-ABS-KEY (((anal OR anus) W/3 (carcinoma* OR adenocarcinoma OR cancer* OR neoplasm* OR tumour* OR tumor* OR malignan* OR squamous OR melanoma OR "small cell")))) AND (TITLE-ABS-KEY (("inflammatory bowel disease*" OR ibd OR crohn* OR "ulcerative colitis")))

Clinialtrials.gov

(anal cancer) AND (non-operative OR nonoperative OR radiotherapy OR radiation OR chemotherapy OR chemo* OR nonsurgical OR non-surgical OR "organ preserv*" OR imrt OR "volumetric modulated arc therapy" OR vmat) AND ("inflammatory bowel disease*" OR ibd OR crohn* OR "ulcerative colitis")

CINAHL

((non-operative OR nonoperative OR radiotherapy OR radiation OR chemotherapy OR chemo* OR nonsurgical OR non-surgical OR "organ preserv*" OR imrt OR "volumetric modulated arc therapy" OR vmat)) AND (((anal OR anus) N3 (carcinoma* OR adenocarcinoma OR cancer* OR neoplasm* OR tumour* OR tumor* OR malignan* OR squamous OR melanoma OR "small cell"))) AND (("inflammatory bowel disease*" OR ibd OR crohn* OR "ulcerative colitis"))
